# Memory-enhancing effects of GEBR-32a, a new PDE4D inhibitor holding promise for the treatment of Alzheimer’s disease

**DOI:** 10.1038/srep46320

**Published:** 2017-04-12

**Authors:** Roberta Ricciarelli, Chiara Brullo, Jos Prickaerts, Ottavio Arancio, Carla Villa, Claudia Rebosio, Elisa Calcagno, Matilde Balbi, Britt T. J. van Hagen, Elentina K. Argyrousi, Hong Zhang, Maria Adelaide Pronzato, Olga Bruno, Ernesto Fedele

**Affiliations:** 1 Department of Experimental Medicine, Section of General Pathology, School of Medical and Pharmaceutical Sciences, University of Genoa, Via L. B. Alberti, 2, 16132 Genoa, Italy; 2 Department of Pharmacy, Section of Medicinal Chemistry, School of Medical and Pharmaceutical Sciences, University of Genoa, Viale Benedetto XV, 3, 16132 Genoa, Italy; 3 Department of Psychiatry and Neuropsychology, School for Mental Health and Neuroscience (MHeNS), Maastricht University, Universiteitssingel 50, 6229 ER Maastricht, The Netherlands; 4 Department of Pathology & Cell Biology, Taub Institute for Research on Alzheimer’s Disease and the Aging Brain, Columbia University, New York, NY 10032, USA; 5 Department of Pharmacy, Section of Pharmacology and Toxicology, School of Medical and Pharmaceutical Sciences, University of Genoa, Viale Cembrano, 4, 16148 Genoa, Italy; 6 Center of Excellence for Biomedical Research, University of Genoa, Viale Benedetto XV, 16132, Genoa, Italy

## Abstract

Memory loss characterizes several neurodegenerative disorders, including Alzheimer’s disease (AD). Inhibition of type 4 phosphodiesterase (PDE4) and elevation of cyclic adenosine monophosphate (cAMP) has emerged as a promising therapeutic approach to treat cognitive deficits. However, PDE4 exists in several isoforms and pan inhibitors cannot be used in humans due to severe emesis. Here, we present GEBR-32a, a new PDE4D full inhibitor that has been characterized both *in vitro* and *in vivo* using biochemical, electrophysiological and behavioural analyses. GEBR-32a efficiently enhances cAMP in neuronal cultures and hippocampal slices. *In vivo* pharmacokinetic analysis shows that GEBR-32a is rapidly distributed within the central nervous system with a very favourable brain/blood ratio. Specific behavioural tests (object location and Y-maze continuous alternation tasks) demonstrate that this PDE4D inhibitor is able to enhance memory in AD transgenic mice and concomitantly rescues their hippocampal long-term potentiation deficit. Of great relevance, our preliminary toxicological analysis indicates that GEBR-32a is not cytotoxic and genotoxic, and does not seem to possess emetic-like side effects. In conclusion, GEBR-32a could represent a very promising cognitive-enhancing drug with a great potential for the treatment of Alzheimer’s disease.

Memory loss characterizes several neurodegenerative pathologies among which Alzheimer’s disease (AD) certainly represents the most common form of dementia. At present, cognitive disorders cannot benefit from effective therapies which are urged, given their socioeconomic impact that is expected to increase dramatically in the near future.

Over the last 30 years, neuroscience research has consistently demonstrated that cyclic adenosine monophosphate (cAMP) and its downstream effectors play a pivotal role in the molecular mechanisms underlying memory formation^1^. Indeed, pharmacological and genetic manipulations aimed at stimulating the cAMP pathway have been shown to enhance cognition under physiological conditions and, more importantly from a translational point of view, to normalize memory in different experimental models of cognitive impairment, including transgenic AD animals.

At the cellular level, it is generally accepted that the pro-cognitive properties of cAMP are due to its key function in the expression of long-term potentiation (LTP), a form of synaptic plasticity that is considered the electrophysiological correlate of memory^2^. As a matter of fact, knockout of adenylyl cyclase, the cAMP-synthesizing enzyme, significantly impairs LTP and memory formation^3^, whereas its overexpression does the opposite^4^,^5^. Similarly, stimulation or blockade of the cAMP effectors protein kinase A (PKA), Exchange Protein Directly Activated by cAMP (EPAC) and cAMP Responsive Element Binding Protein (CREB), respectively facilitates or disrupts LTP and memory^6^,^7^,^8^,^9^,^10^,^11^,^12^,^13^,^14^,^15^,^16^,^17^.

A large body of evidence also indicates that inhibition of the phosphodiesterase (PDE)-mediated hydrolysis of cAMP could represent a successful therapeutic strategy to treat memory deficits. Among the 11 different PDEs, the type 4 family (PDE4) has been identified as one of the most promising target for the treatment of cognitive-related disorders^18^,^19^,^20^,^21^,^22^.

The PDE4 family comprises four isoforms (PDE4A-D), but pan-PDE4 inhibitors, such as rolipram, albeit being effective pro-cognitive drugs in pre-clinical settings, are endowed with severe undesired side effects (i.e. emesis) that have hampered their clinical use^23^.

Recently, PDE4D has emerged as a specific molecular target to develop selective inhibitors having positive effects on memory and improved side-effect profile^24^. In this context, our group has recently synthesized and characterized several selective PDE4D full inhibitors, some of which showed cognitive-enhancing properties in rodents at doses that were devoid of emetic-like effects^25^,^26^,^27^,^28^,^29^,^30^.

In line with lead optimization processes, we here report the development of the novel PDE4D full inhibitor GEBR-32a, a compound that exhibits improved brain and cell penetration and that is able to efficiently increase cAMP levels, to rescue impaired hippocampal LTP and to improve memory function in normal and AD mice. Importantly, GEBR-32a has no cytotoxic or genotoxic potential and does not evoke emetic-like effects.

## Results

### Synthesis and enzymatic profile of GEBR-32a

**Figure 1:**
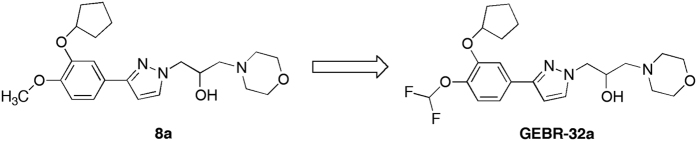
Chemical structure of compounds 8a and GEBR-32a.

GEBR-32a was designed by our group as a fluorinated derivative of the lead compound 8a^30^ (Fig. 1). The 4-(difluoromethoxy)-3-hydroxybenzaldehyde, a key intermediate for GEBR-32a synthesis, was prepared using a novel microwave assisted procedure^29^ with improved yield with respect to other protocols reported in the literature.

GEBR-32a selectivity was evaluated on a panel of 20 recombinant human PDE isoforms and variants. At the concentration of 10 μM, GEBR-32a was devoid of any significant activity toward PDE1B, 2A3, 4A4,B2, 5A1, 7A,B, 8A1,B1, 9A1, 10A1 and 11A1, whereas it showed some inhibitory effect on PDE4A1,B1,B3 (Table 1).

On the other hand, our compound was very active on all the PDE4D variants analysed (Table 1) that were inhibited by more than 50%. The calculated IC_50s_ of GEBR-32 towards those variants ranged from 1.16 to 4.97 μM (Table 2).

### Effect of GEBR-32a on intracellular cAMP levels

**Figure 2:**
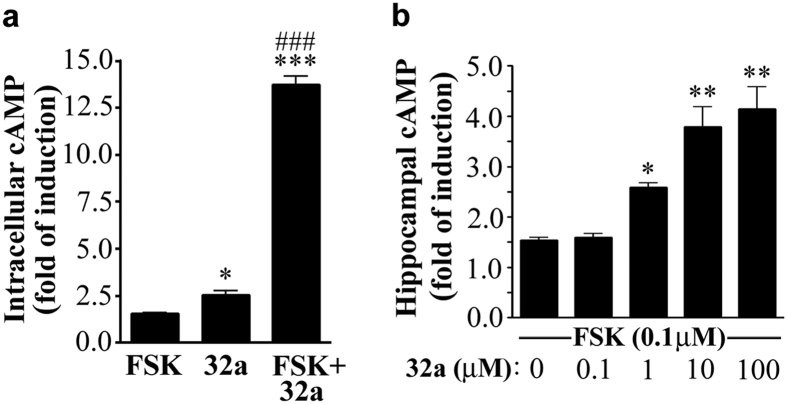
Effect of GEBR-32a on cAMP levels. (**a**) Quantification of cAMP in HTLA cells. Samples were pre-treated for 10 min with GEBR-32a (32a; 100 μM) or an equal volume of DMSO. Then, 1 μM forskolin (FSK) was added, where indicated, for 20 min. The histogram shows the mean ± s.e.m. for three independent experiments; ***P < 0.001 and *P < 0.05 vs vehicle treated cells; ^###^*P* < 0.001 vs FSK alone (ANOVA and post hoc Newman-Keuls test. (**b**) Quantification of cAMP in rat hippocampal slices. Slices (n = 7 per condition) were pre-treated with increasing amount of GEBR-32a (32a) for 15 min and then stimulated with 0.1 μM forskolin for 15 min. The histogram shows the mean ± s.e.m. for six independent experiments; *P < 0.05 and **P < 0.01 vs samples receiving forskolin alone (ANOVA and post hoc Dunnett’s test).

When neuronal HTLA cells were treated with GEBR-2a (100 μM), a 2.5 fold increase of cAMP was observed with respect to controls, and a more marked effect was measured in the presence of the adenylyl cyclase activator forskolin (1 μM) (Fig. 2a).

In rat hippocampal slices, GEBR-32a (0.1–100 μM) was able to increase the forskolin (0.1 μM)-induced cAMP production with a 4-fold elevation observed at the highest concentration tested and an apparent EC_50_ of 1.80 μM (Fig. 2b).

### Safety and pharmacokinetic profile of GEBR-32a

**Figure 3:**
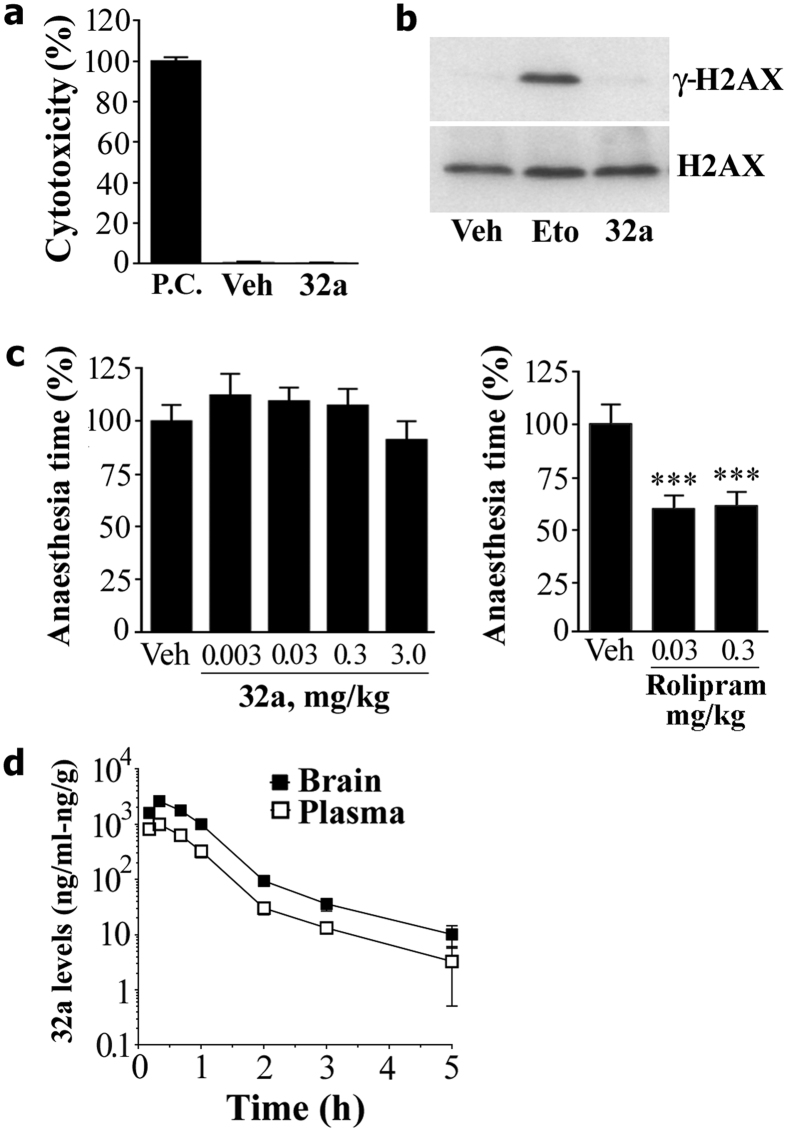
Safety and pharmacokinetic profile of GEBR-32a. (**a**) Cytotoxic potential of GEBR-32a (32a; 100 μM) or DMSO (Veh), relative to the positive control (P.C.), in HTLA cells. Data represent the mean ± s.e.m. of three independent experiments. (**b**) Western blot analysis of γ-H2AX in HTLA cells treated for 24 h with 100 μM of the genotoxic compound etoposide (Eto), GEBR-32a (32a) or an equal volume of DMSO (Veh). The H2AX signals represent the internal loading control. The image shows cropped blots and is representative of three independent experiments all showing essentially similar results. (**c**) Ketamine/xylazine induced α_2_-adrenoceptor-mediated anaesthesia test as a measure of emetic-like effect. GEBR-32a (n = 10–12 per group) or Rolipram (5–7 per group) were administered (s.c.) 15 min after the induction of anesthesia. Displayed are the mean times (±s.e.m.) the mice stayed anesthetized during every condition, relative to vehicle (Veh, set at 100%). Data for Rolipram have been redrawn from ref. 26. ***p < 0.001 vs vehicle (One-way ANOVA followed by Bonferroni’s comparison t-test). (**d**) Pharmacokinetic analysis of GEBR-32a. The graph shows the plasma/brain concentrations of GEBR-32 over time following the subcutaneous injection of a 10 mg/kg dose in BALB/c mice. Each point represents the mean (±s.e.m.) of three determinations.

In HTLA cells, lactate dehydrogenase release and the phosphorylation of the chromatin-bound histone HA2.X (a marker of DNA damage) were analysed to assess cyto- and genotoxicity, respectively. As shown in Fig. 3a,b, 24 hours of exposure to a single high concentration (100 μM) of GEBR-32a did not produce any significant effect in both assays.

The emetic-like effects of GEBR-32a were investigated using the xylazine/ketamine-induced anaesthesia test, which indicated that the administration of GEBR-32a (0.003–3 mg/kg) did not significantly influence the duration of anaesthesia in adult mice (Fig. 3c). Under the very same experimental conditions, in our previous study the pan PDE4 inhibitor rolipram significantly shortened anaesthesia time already at 0.03 and 0.3 mg/kg^26^ (Fig. 3c).

As for the pharmacokinetic, GEBR-32a (10 mg/kg) was rapidly absorbed and distributed to the brain (T_max_ = 20 min) and also rapidly eliminated with a half-life of approximately 1 h (Fig. 3d and Table 3). The brain to plasma AUC_0-t_ ratio was 2.71, indicating a favourable brain penetration of our PDE4D inhibitor.

### Effects of GEBR-32a in the object location task (OLT) in wild type and Tg2576 mice

**Figure 4:**
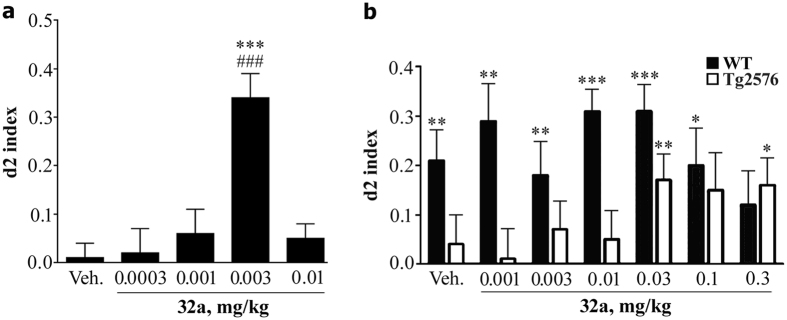
Acute administration of GEBR-32a improves memory consolidation in the object location test. The figure reports the d2 index (see Methods) measured under different experimental conditions. (**a**) GEBR-32a (32a) or vehicle (Veh) were administered to healthy adult mice 3 hours after the learning trial T1 and the test trial T2 was performed 24 hours after T1. d2 index is reported in the figure. Each bar represents mean (±s.e.m.) of 22–24 mice. ^###^P < 0.0001 vs. zero (chance level; one-sample t-test); ***P < 0.001 vs. vehicle (post-hoc; Dunnett’s test). (**b**) GEBR-32a or vehicle were administered to aged WT or Tg2576 mice 30 min before the learning trial T1 and the test trial T2 was performed 1 hour after T1. Each bar represents mean (±s.e.m.) 13–16 mice. *P < 0.05, **P < 0.01, ***P < 0.001 vs. zero (chance level; one-sample t-test).

Figure 4a reports the performance of adult WT mice in the OLT, following administration of vehicle or of GEBR-32a (0.0003–0.01 mg/kg) with an inter-trial interval of 24 hours. With this long-time interval, vehicle-treated mice did not remember the disposition of the objects they had visited in the learning trial T1 and, therefore, they spent almost the same time in exploring the two objects during the test trial T2. Thus, their d2 index was not different from zero. On the contrary, when mice were treated with GEBR-32a at the dose of 0.003 mg/kg, 3 hours after the learning trial T1, they did recognize that one object had been moved from its original position and, during the test trial T2, they explored it for much more time than the object that had not been moved. Therefore, their d2 index was different from zero.

Figure 4b summarizes the results in the OLT, following administration of GEBR-32a (0.001–0.3 mg/kg), but with an inter-trial interval of 1 hour. With this short-time interval, control aged WT animals (vehicle) with intact memory were able to remember the position of the two objects and, therefore, during the test trial T2 they explored the moved object more than the unmoved one, thus showing a d2 index > 0. On the contrary, age-matched vehicle-treated Tg2576 mice did not. Administration of GEBR-32a to aged WT animals did not increase their normal discrimination capabilities, whereas Tg2576 mice showed a significant improvement in memory performance, the d2 index being higher than zero at the doses of 0.03 and 0.3 mg/kg.

### Effects of GEBR-32a in the Y-maze continuous alternation task in wild type and Tg2576 mice

**Figure 5:**
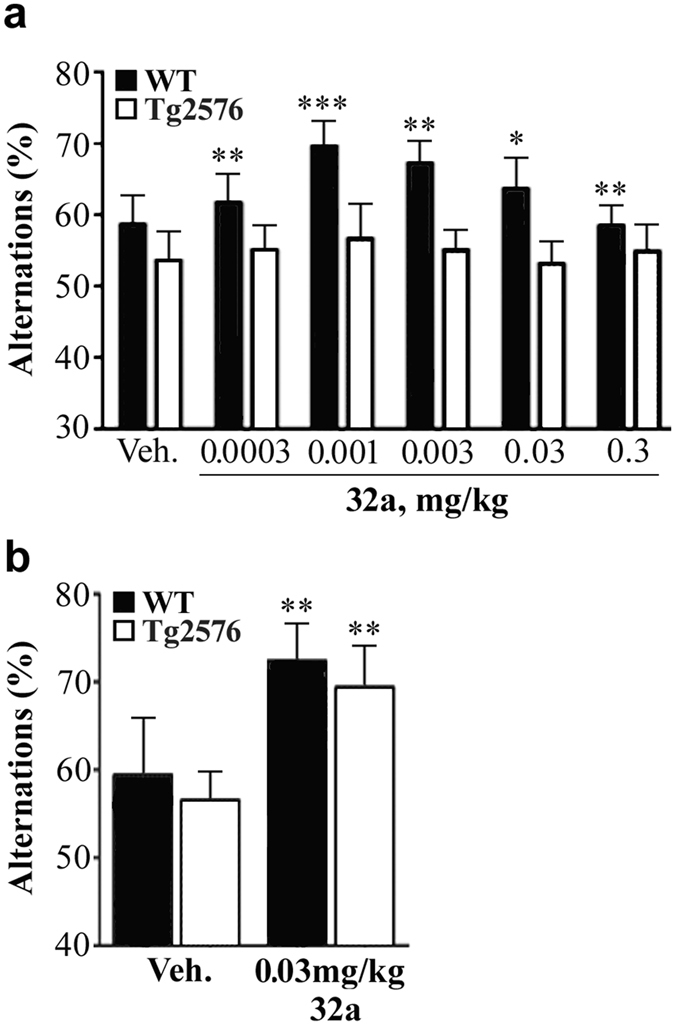
Acute and chronic effects of GEBR-32a in the Y-maze continuous alternation test. The figure reports the average percentage of alternations (see Methods) measured under different experimental conditions. (**a**) GEBR-32a (32a) or vehicle (Veh) were acutely administered to aged WT or Tg2576 mice 30 min before the trial. Each bar represents mean (±s.e.m.) of 5–10 mice. *P < 0.05, **P < 0.01, ***P < 0.001 vs. 50% alternations (chance level; one-sample t-test). (**b**) GEBR-32a was administered chronically (s.c.) for up to 23 days before the trial. Each bar represents mean (±s.e.m.) of 9–10 mice. **P < 0.01 vs 50% alternations (chance level; one-sample t-test).

The effects of GEBR-32a administration (0.0003–0.3 mg/kg) on Y-maze alternation performance in aged WT and Tg2576 mice are summarized in Fig. 5a. When treated with vehicle, both aged WT and transgenic mice did not show a performance significantly higher than chance level (50% alternations), indicating that under these test conditions they were not able to correctly remember the arms already visited. GEBR-32a significantly ameliorated the performance in control animals at all the doses tested (alternations always higher than 50%). Under the same conditions, GEBR-32a was not able to induce any improvement in Tg2576 mice. However, when the PDE4D inhibitor was chronically administered (0.03 mg/kg for up to 23 days), also the transgenic animals showed alternations significantly higher than 50% (Fig. 5b), indicating amelioration of memory function.

### Effect of GEBR-32a on hippocampal long-term potentiation in wild type and Tg2576 mice

**Figure 6:**
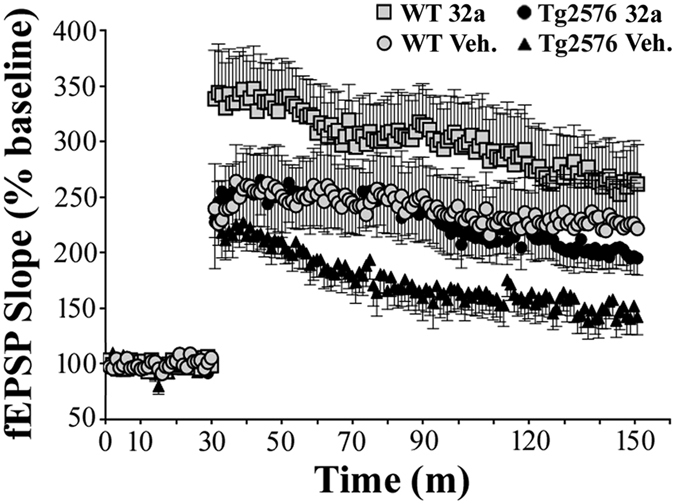
Chronic administration of GEBR-32a rescues hippocampal long-term potentiation deficits in Tg2576 mice. WT and Tg2576 mice were treated with vehicle (Veh) or GEBR-32a (32a; 0.03 mg/kg) for 23 days. Electrophysiological recordings of field excitatory post-synaptic potentials (fEPSP) have been carried out on fresh hippocampal slices obtained from the respective mice and are reported in the figure as percentage of the mean baseline value (defined 100%) that was determined by averaging the recordings of the first 30 min. LTP was evoked as reported in the Methods. Each point represents mean (±s.e.m.). Statistical analysis demonstrated that Tg2576 mice exhibited a significant impairment in comparison to aged-matched WT mice (2 hours LTP: WT veh vs. Tg2576 veh F_1,18_ = 4.942, p = 0.0393; last 10 min LTP: WT veh vs. Tg2576 veh F_1,18_ = 5.672, p = 0.0285). Chronic treatment with GEBR-32a was able to significantly restore normal LTP in Tg2576 mice (2 hours LTP: Tg2576 veh vs. Tg2576 32a F_1,18_ = 3.651, P = 0.0721; last 10 min LTP: Tg2576 veh vs. Tg2576 32a: F_1,18_ = 6.014, P = 0.0246).

As shown in Fig. 6, vehicle-treated Tg2576 mice displayed a significant LTP impairment in comparison with age-matched WT controls. In the latter, chronic GEBR-32a administration (0.03 mg/kg for 23 days) caused a clear, though not significant, enhancement of LTP in comparison with respective controls. Of great relevance, GEBR-32a was able to rescue the LTP deficit of Tg2576 mice to the level of vehicle-treated WT animals.

## Discussion

In this study, we present GEBR-32a, a novel PDE4D inhibitor deriving from the lead optimization process of compound 8a, which was recently synthesized and characterized in our laboratories^30^.

A key strategy in modern drug design is to optimize absorption, distribution, metabolism and excretion (ADME) before a drug enters the clinical phase. In particular, drugs designed for neurodegenerative disorders need to readily cross the blood-brain barrier (BBB) in order to reach their targets. In pharmaceutical chemistry, introduction of fluorine atoms represents a common strategy to improve drug potency and optimize ADME parameters^31^,^32^,^33^,^34^. Actually, the number of fluorinated drugs approved by FDA and EMA has greatly increased over the last decade^35^. Fluorine substitution influences different aspects of drug-target interaction, as well as of ADME, by modulating basic or acid molecule pKa, structure conformation, hydrophobic interactions, lipophilicity and interaction with metabolizing enzymes^31^,^32^,^33^,^34^,^35^.

Our selectivity analysis, carried out on a panel of 20 PDE isoforms and variants, has confirmed that GEBR-32a is very active on PDE4D isoforms and its potency has been indeed improved in comparison to its parent compound 8a. For example, GEBR-32a showed an IC_50_ of 2.43 μM toward PDE4D3, whereas that of compound 8a was 7.60 μM^30^. Fluorine introduction had even more dramatic effects on BBB permeability, the brain/plasma ratio being extremely improved from 0.76 for compound 8a to 2.71 for GEBR-32a. For comparison, this ratio for the well-known PDE4 inhibitors rolipram and roflumilast is 2 and 1, respectively^36^,^37^.

In addition, the presence of two fluorine atoms did not alter the ability of GEBR-32a to functionally inhibit PDE4D enzymes, as demonstrated by the significant elevation of intracellular cAMP levels observed *in vitro*, in both cultured cells and hippocampal slices (Fig. 2). Of note, 100 μM GEBR-32a was able to cause a 12-fold increase of the forskolin-induced elevation of cAMP in cultured cells, while the same concentration of its parent compound 8a evoked only a 4-fold increase under the very same experimental conditions^30^, indicating a better cell membrane diffusion of the fluorinated derivative.

Although some natural occurring fluorine-containing molecules are highly toxic (e.g. sodium monofluoroacetate), it is now well known that introduction of fluorine into drugs does not necessarily confer toxicity and, on the contrary, may reduce the toxicity of parent compounds by preventing their metabolic bioactivation^31^,^35^. As a matter of fact, our preliminary toxicological screening demonstrated that GEBR-32a does not have either cytotoxic or genotoxic effects, even when cells were exposed to a high concentration of the drug (100 μM) for 24 hours (Fig. 3a,b).

As inhibition of PDE4D activity has been consistently shown to ameliorate memory formation^26^,^28^,^30^,^38^,^39^, GEBR-32a was trialled in two different behavioural tests, the object location task (OLT) and the Y-maze alternation task. To verify that fluorine introduction had not influenced the pro-cognitive properties of the parent compound 8a in normal adult mice^30^, we tested GEBR-32a under the same experimental conditions.

The OLT is used to assess episodic-like spatial memory by evaluating if mice recognize/remember that one of two objects, explored in the learning trial, has been moved to a different position in the test trial. In this case, vehicle-treated adult mice did not remember the spatial arrangement of the objects when tested in the OLT with an inter-trial delay of 24 hours, due to natural forgetting (Fig. 4a). On the contrary, when mice were treated with GEBR-32a, at a very low dose (0.003 mg/kg) three hours after the learning trial, a time point at which PDE4 inhibitors specifically improve memory consolidation^40^, they were able to remember that one object was not in the same position as in the learning trial and, consequently, spent much more time in exploring it in the test trial (d2 index > 0). Therefore, GEBR-32a is indeed able to improve consolidation of spatial long-term memory 3 h after learning.

However, the observation that a drug improves cognition in normal animals, although of interest for drug development, has limited relevance from a translational point of view. For this reason, we further analysed the memory-enhancing properties of GEBR-32a on aged Tg2576 mice that are widely used to model Alzheimer’s dementia. When these mice are 9–12 months old, they show ß-amyloid plaque accumulation in brain tissue and synaptic and memory deficits^41^.

In these experiments, we evaluated the effects of GEBR-32a in the OLT with a 1-hour inter-trial interval, which allows normal animals to retain the information acquired during the learning trial. In this paradigm, in fact, vehicle-treated aged WT mice showed well-functioning short-term spatial memory (Fig. 4b), as they identified that one object had been moved from its original position and, therefore, they explored it for more time (d2 index > 0). For this reason, administration of GEBR-32a had no effects on their performance, as short-term memory cannot be further improved probably due to ceiling effect. On the contrary, a memory impairment was evident in vehicle-treated Tg2576 mice, which were unable to distinguish the moved object and their d2 index was, in fact, not different from zero. Of great relevance, when these transgenic mice were acutely treated with GEBR-32a, they showed a relevant improvement of their short-term memory performance, exploring the moved object for more time during the test trial.

The Y-maze continuous alternation task analyses spatial working memory by measuring the number and the order of entries of mice into the three different arms of the maze. In this case, a 50% alternation is considered chance level and reflects the absence of working memory performance. Indeed, adult WT mice had a performance compatible with intact working memory (alternations higher than 50%), which was not further improved by GEBR-32a administered 30 min before the trial (ceiling effect; data not shown). On the other hand, both aged WT and Tg2576 mice manifested memory impairment, since they scored alternations not different from chance level when treated with vehicle (Fig. 5a). When GEBR-32a was acutely administered 30 min before the test, aged WT mice had a significant memory improvement, whereas aged Tg2576 mice had not, suggesting that an acute increase of cAMP is not sufficient to ameliorate this type of cognitive deficit in this mouse model of Alzheimer’s disease. However, if chronically treated with GEBR-32a, also Tg2576 mice performed significantly better than chance levels. These results demonstrate that the impaired working memory can be recovered by a chronic treatment with the selective PDE4D inhibitor.

In the attempt to correlate GEBR-32a memory-enhancing properties with cellular/molecular mechanisms, we investigated the effects of GEBR-32a on hippocampal long-term potentiation (LTP). Hippocampal LTP is a form of synaptic plasticity that is generally accepted to represent the molecular substrate of memory formation and is known to require a fine tuning of the cAMP/PKA/CREB pathway to function^24^. Moreover, a large body of evidence has shown that PDE4 inhibitors are able to rescue compromised LTP in different models of pathological conditions, including AD^42^,^43^.

Using GEBR-32a, we demonstrate for the first time that chronic inhibition of PDE4D is able to rescue the LTP deficit that is observed in Tg2576 mice (Fig. 6). This supports the notion that, among the different PDE4 isoforms, PDE4D is critically involved in plasticity, learning and memory processes^21^,^44^.

To date, the serious emesis associated with non isoform-selective PDE4 inhibitors has impeded their clinical exploitation as promnesic drugs in those pathologies, such as AD, characterized by memory loss. Although rodents cannot vomit, it has been shown that preliminary screening for emetic-like potential of PDE4 inhibitors can be reliably carried out in these animals using the xylazine/ketamine-induced anaesthesia test^45^. It has been proposed that PDE4 inhibitors cause emesis by activating a sympathetic pathway through antagonism of presynaptic α_2_-adrenergic receptors, a hypothesis supported by the evidence that α_2_-adrenergic antagonists stimulate vomiting, while clonidine, a selective α_2_-receptor agonist, is able to prevent PDE4 inhibitor-induced emesis. In addition, α_2_-adrenergic antagonists, as well as PDE4 inhibitors, reduce the time of anaesthesia induced by a xylazine/ketamine mixture^45^,^46^. Using this test, we have recently confirmed that rolipram is able to reduce the anaesthesia time at the same dose improving memory in mice^26^. On the contrary, our initial hits^26^,^30^ and our optimized lead GEBR-32a (Fig. 3c) are ineffective on anaesthesia time at doses 100–1000 times higher than the pro-cognitive ones, thus indicating that they are very likely devoid of emetic side-effects.

Therefore, the memory-improving effects of GEBR-32a are further dissociated from possible emetic-like effects, as compared to its parent compound 8a. A possible explanation could be that GEBR-32a (as 8a) inhibits PDE4D isoforms involved in memory processes per se. As for inhibition mechanisms, our relatively high IC_50_ values reflect binding of GEBR-32a to the catalytic domain, yet interactions outside the catalytic domain cannot be ruled out, resulting in more or less selectivity for specific PDE4D isoforms. Clearly, more research is needed into this aspect, including co-crystallization of GEBR-32a and PDE4D isoforms to elucidate their interactions. Moreover, we need to know which specific PDE4D isoform to target for the treatment of memory decline in Alzheimer’s disease^47^.

As for the molecular mechanisms underlying the pro-cognitive effects of GEBR-32a in AD mice, it can be hypothesised that our PDE4D inhibitor reverses the Aβ-mediated inhibition of the cAMP/PKA/CREB pathway, as it happens with rolipram^42^,^48^, thus leading to the rescue of hippocampal LTP and memory deficits. Moreover, it has been reported that PDE4 inhibition can also reduce neuroinflammation by dampening the production of the proinflammatory cytokines IL-1b and TNFα and by increasing the levels of TGFβ-1 and BDNF, which are known to play a key role in hippocampal synaptic plasticity and memory^49^,^50^,^51^.

In conclusion, GEBR-32a is a selective PDE4D inhibitor endowed with a very favourable toxicological and pharmacokinetic profile, and is able to improve spatial memory processes without undesired emetic-like side effects. Although further safety studies are needed, GEBR-32a is a promising therapeutic agent for the treatment of cognitive decline in AD and related dementias.

## Methods

### Synthesis and enzymatic profile of GEBR-32a

**Figure 7:**
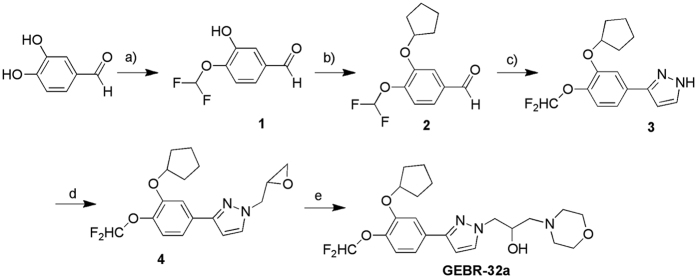
Synthesis of GEBR-32a. Reagents and Conditions: a) CClF_2_COOCH_3_, Cs_2_CO_3,_ anydrous DMF, 300 W, 90 °C, 25 min., yield 57%; b) bromocyclopentane, K_2_CO_3_/KI, an. DMF, 65 °C, 22 h, yield 87%; c) p-toluenesulfonyl hydrazide, anhydrous acetonitrile, RT, 1 h; 5N NaOH solution RT, 20 min.; 1-vinylimidazole, 50 °C, 48 h, yield 45%; d) epichlorohydrin 0–5 °C; then, TEA, stirred until 25 °C, then 70 °C, 6 h, 58%; e) morpholine excess, 50–60 °C, 18 h, yield 56%.

Figure 7 reports the synthesis of GEBR-32a. We prepared the starting product 4-(difluoromethoxy)-3-hydroxybenzaldehyde (**1**) using our recently reported microwave assisted procedure with improved yield in comparison to many other procedures reported in the literature^29^. The 3-(cyclopentyloxy)-4-(difluoromethoxy)benzaldehyde (**2**) was then obtained by alkylation with bromocyclopentane in anhydrous *N,N*-dimethylformamide (DMF), in the presence of potassium carbonate and potassium iodide [A. Thomas *et al*. PCT Int. Appl., 2004, WO 2004016596]. Then, we prepared the 3-[3-(cyclopentyloxy)-4-(difluoromethoxy)phenyl]-1*H*-pyrazole (**3**) by one-pot 1,3-dipolar cycloaddition between the benzaldehyde (**2**) and p-toluenesulfonyl hydrazide, and subsequent addition of NaOH and 1-vinylimidazole in acetonitrile. The pyrazole intermediate (**3**) was then treated with an excess of epichlorohydrin in the presence of TEA to obtain the 3-[3-(cyclopentyloxy)-4-(difluoromethoxy)phenyl]-1-(oxiran-2-ylmethyl)-1*H*-pyrazole (**4**) as a single isomer. Finally, GEBR-32a was obtained by reacting the oxirane (**4**) with an excess of morpholine.

#### Synthesis of 3-[3-(cyclopentyloxy)-4-(difluoromethoxy)phenyl]-1H-pyrazole

A solution of 3-(cyclopentyloxy)-4-(difluoromethoxy)benzaldehyde **2** (1.65 g, 6.45 mmol) in anhydrous acetonitrile (2 mL) was added to p-toluenesulfonyl hydrazide (1.2 g, 6.45 mmol) solved in anhydrous acetonitrile (7 mL) and the mixture was stirred at room temperature for 1 h. Then, 5 N NaOH solution (1.29 mL, 6.45 mmol) was added and the mixture, which became coloured in red, was stirred at room temperature for 20 min. Afterward, 1-vinylimidazole (3.04 g, 32.25 mmol, 2.92 mL) was slowly added and the mixture was heated at 50 °C for 48 h. After cooling to room temperature, the solvent was removed under reduced pressure and the crude was solved in ethylacetate (10 mL). The organic phase was washed with brine (2 × 10 mL), 1 N HCl solution (10 mL), water (3 × 10 mL), dried (MgSO_4_), and concentrated under reduced pressure to afford a light yellow oil which was purified by silicagel (100–200 mesh) column chromatography using CH_2_Cl_2_ as eluent. The pure product was obtained as a light yellow oil. Yield: 45%. ^1^H-NMR (CDCl_3_): ^δ^1.52–1.94 (m, 8H, 4CH_2_ cyclopent.), 4.80–5.00 (m, 1H, OCH cyclopent.), 6.55–7.70 (m, 7H, 3H Ar + H-5 pyraz. + H-4 pyraz + OCHF_2_ + NH pyraz.). Anal. (C_15_H_16_N_2_O_2_F_2_) C, H, N. (% calculated/found) C: 61.22/61.32; H: 5.48/5.71; N: 9.52/9.59.

#### Synthesis of 3-[3-(cyclopentyloxy)-4-(difluoromethoxy)phenyl]-1-(oxiran-2-ylmethyl)-1H-pyrazole

A mixture of 3-[3-(cyclopentyloxy)-4-methoxyphenyl]-1*H*-pyrazole **3** (1.3 g, 4.42 mmol) and epichlorohydrin (4.5 mL, 57.77 mmol) was cooled at 5 °C. Afterward, TEA (6.63 mmol, 0.92 mL) was added dropwise and the reaction mixture was stirred until the temperature became 25 °C. Then, the mixture was heated at 70 °C for 6 h. After cooling to room temperature, the mixture was poured into water (100 mL), the aqueous phase was extracted with CH_2_Cl_2_ (2 × 20 mL). The organic phase was washed with water (20 mL), until the pH of the washing solution became neutral, and with brine (3 × 20 mL). It was, then, dried (MgSO_4_) and concentrated under reduced pressure, yielding an oil that was purified by silicagel (100–200 mesh) column chromatography using diethyl ether as eluent. The pure product was obtained as a light yellow oil. Yield: 58%. ^1^HNMR (CDCl_3_): ^δ^1.58–2.04 (m, 8H, 4CH_2_ cyclopent.), 2.53–2.61 (m, 1H, H_A_ of CH_2_ epox.), 2.83–2.94 (m, 1H, H_B_ of CH_2_ epox.), 3.36–3.48 (m, 1H, CHO epox.), 4.13–4.28 and 4.52–4.65 (m, 2H, CH_2_N pyraz), 4.93–5.03 (m, 1H, OCH cyclopent.), 6.56 (d, *J* = 2.4 Hz, 1H, H-4 pyraz.), 6.58 (t, *J* = 75.6, 1H, OCHF_2_), 7.19 (d, *J* = 8.0 Hz, 1H, H-5 Ar), 7.29 (dd, *J* = 8.0, 2.0 Hz, 1H, H-6 Ar), 7.48 (d, *J* = 2.0 Hz, 1H, H-2 Ar), 7.53 (d, *J* = 2.4 Hz, 1H, H-5 pyraz.). Anal. (C_18_H_20_N_2_O_3_F_2_) C, H, N. (% calculated/found) C: 61.71/61.86; H: 5.75/5.99; N: 8.00/8.07.

#### Synthesis of 1-{3-]3-(cyclopentyloxy)-4-(difluoromethoxy)phenyl]-1H-pyrazol-1-yl}-3-morpholin-4-ylpropan-2-ol (GEBR-32a)

An excess of morpholine (1 mL) was added to 3-[3-(cyclopentyloxy)-4-(difluoromethoxy)phenyl]-1-(oxiran-2-ylmethyl)-1*H*-pyrazole **7** (0.37 g, 1.06 mmol) and the mixture was heated at 50–60 °C for 18 h. After cooling to room temperature, the mixture was diluted with diethyl ether (20 mL), then the organic phase was washed with water (20 mL), dried (MgSO_4_) and concentrated under reduced pressure. The crude was purified by silicagel (100–200 mesh) column chromatography using, as eluents, diethyl ether first and then a mixture of CH_2_Cl_2_/CH_3_OH (9:1) to obtain the pure product as light yellow oil.

Yield: 56%.^1^H-NMR (CDCl_3_): ^δ^1.60–2.07 (m, 8H, 4CH_2_ cyclopent.), 2.44–2.84, 3.63–3.93 (2 m, 10H, 4CH_2_ morph. + CH_2_N), 4.13–4.40 (m, 3H, CH_2_N pyraz. + *CH*-OH), 4.82–4.92 (m, 1H, OCH cyclopent), 6.54 (d, *J* = 2.4, 1H, H-4 pyraz.), 6.58 (t, *J* = 75.6 Hz, 1H, OCHF_2_), 7.18 (d, *J* = 8.0 Hz, 1H, H-5 Ar), 7.28 (d, *J* = 8.0 Hz, 1H, H-6 Ar), 7.44 (s, 1H, H-2 Ar), 7.56 (d, *J* = 2.4, 1H, H-5 pyraz.). IR (CHCl_3_) cm^−1^: 3413 (OH). Anal. (C_22_H_29_N_3_O_4_F_2_) C, H, N. (% calculated/found) C: 60.40/60.40; H: 6.68/6.81; N: 9.61/9.24.

All chemicals were purchased from Chiminord and Aldrich Chemical (Milan, Italy). Solvents were reagent grade. Unless otherwise reported, all commercial reagents were used without any further purification. Aluminium backed silica gel plates (Merck DC-Alufolien Kieselgel 60 F254, Darmstad, Germany), were used in thin-layer chromatography (TLC) for routine monitoring the course of reactions. Detection of spots was made by UV light. Merck silica gel, 230–400 mesh, was used for chromatography.

Melting points are not “corrected” and were measured with a Buchi M-560 instrument. IR spectra were recorded with a Perkin-Elmer 398 spectrophotometer. ^1^H NMR spectra were recorded on a Varian Gemini 200 (200 MHz) instrument, chemical shifts are reported as δ (ppm) relative to tetramethylsilane (TMS) as internal standard; signals were characterized as s (singlet), d (doublet), t (triplet), q (quartet), m (multiplet), br s (broad signal); J in Hz. Elemental analyses were determined with an elemental analyzer EA 1110 (Fison-Instruments, Milan, Italy) and the purity of all synthesized compounds was >95%.

The enzymatic profile of GEBR-32a has been carried out by Scottish Biochem (Glasgow, Scotland, UK), using recombinant human PDE4 enzymes expressed in baculovirus, as previously reported^25^. In a first assay, GEBR-32a was preliminary tested at a single concentration (10 μM) in duplicate on a panel of 20 PDE isoforms and variants; then, IC_50_ values were determined only for those PDEs whose inhibition by 10 μM GEBR-32a was more than 50%. The compound was tested at five different concentrations (1 nM to 100 μM) and the IC_50s_ were obtained by non-linear regression analysis of the concentration-inhibition curves (GraphPad Prism software).

### Animals

C57BL/6J mice were supplied by Charles River (Sulzfeld, Germany) to Maastricht University. Tg2576 and wild type (WT) mouse colonies were maintained in the animal facilities of the Columbia University. The correct genotype was identified by PCR on tail samples. Adult Sprague-Dawley rat colony was maintained in the animal facilities of the Department of Pharmacy, University of Genoa. Only male animals were used in the experiments.

For behaviour experiments, adult (3–4 months of age) WT, aged WT and Tg2576 mice (12–21 months of age) were housed individually on a reverse light/dark cycle (light from 19:00 to 07:00). Rats were housed on a regular light/dark cycle (light from 07:00 to 19:00). Animals were kept at constant temperature (22 ± 1 °C) and relative humidity (50%) with free access to food and water.

All the respective experimental procedures have been approved by the IACUC Ethics Committee of Columbia University (USA), the Ethics Committee of Maastricht University (NL) and the Italian Ministry of Health for the University of Genoa (IT). All animal experiments have been carried out in accordance with the EU Directive 2010/63/EU for animal experiments and the National Institutes of Health guide for the care and use of Laboratory animals (NIH Publications No. 8023, revised 1978). All efforts were made to minimize animal suffering and to use the minimal number of animals to produce reliable results.

### cAMP determination in cultured cells and hippocampal slices

The cells used in this study (human neuroblastoma cell line HTLA-230, HTLA) were grown in Roswell Park Memorial Institute medium (RPMI), with 0.1 mM non-essential aminoacids and 10% fetal bovine serum. At the end of treatments, intracellular cAMP was measured with the DetectX^®^ Direct Cyclic AMP Enzyme Immunoassay Kit (Arbor Assay, MI, USA), following the manufacturer’s protocol.

Hippocampal slices (400 μm thick) were obtained from adult Sprague Dawley rats using a McIlwain tissue chopper and pre-incubated in physiological medium^52^ (7 slices/condition) at 37 °C for 15 min, first with different concentrations of GEBR-32a and, then, with 0.1 μM forskolin for further 15 min. Finally, slices were left in hot medium (90 °C) for 15 min, sonicated and centrifuged at 10000 g for 15 min. The supernatant was used for cAMP determination, using the same kit as above, and the pellet for protein quantification.

Results from cells and slices are presented as fold increase and represent mean ± S.E.M. of the number of experiments reported in the figure legend. Normal distribution was evaluated by Kolmogorov–Smirnov test and then data have been analysed by one-way ANOVA followed by Newman-Keuls or Dunnett’s tests, as appropriate.

### Cytotoxicity and genotoxicity in cultured cells

For the cytotoxicity and genotoxicity assays, HTLA cells were treated for 24 hours with GEBR-32a (100 μM) at 37 °C. At the end of the incubation period, lactate-dehydrogenase release was measured in conditioned media using the Cytoxicity Detection Kit^PLUS^ (Roche, Germany) according to manufacturer protocols.

To evaluate genotoxicity, cells were processed for total protein extraction as described previously^53^. Immunoblots were done according to standard methods, using the following antibodies: mouse monoclonal to gamma H2A.X (2F3, phospho S139) and rabbit polyclonal to Histone H2A.X (Abcam, UK); anti-mouse and anti-rabbit secondary antibodies coupled to horseradish peroxidase (GE Healthcare, UK). Proteins were visualized with an enzyme-linked chemiluminescence detection kit according to the manufacturer’s instructions (GE Healthcare). Chemiluminescence was monitored by exposure to films and signals were analyzed under non-saturating condition with an image densitometer (Bio-Rad, CA, USA).

### Pharmacokinetic analysis

A total of 21 male BALB/c mice were used for each drug and three mice were used for each time point^30^. GEBR-32a was dissolved in DMSO, diluted in 0.5% methylcellulose to yield a final concentration of 1 mg/mL and administered subcutaneously at the dose of 10 mg/kg. Blood samples (approximately 250 *μ*L) were collected via retro-orbital puncture into tubes containing sodium heparin at 10 min, 20 min, 40 min, 1 h, 2 h, 3 h, and 5 h post-injection. Plasma was separated by centrifugation (11000 rpm, 5 min, 4 °C) and stored at −70 °C before analysis. After blood harvest, mice were sacrificed by cervical dislocation and brains were excised, weighed, rinsed by cold saline and then frozen at −70 °C until analysis.

For blood analysis, 25 *μ*L of plasma were transferred into a 1.5 mL Eppendorf tube and were added with 25 *μ*L of methanol and 25 *μ*L of internal standard (500 ng/mL voriconazole), followed by the addition of 100 *μ*L methanol.

As for brains, 100 mg of tissue were placed into a plastic tube and added with 500 *μ*L of methanol to facilitate homogenization, which was carried out using a Fluko F6/10 superfine homogenizer for approximately 1 min. Homogenized samples were then treated by ultrasound for 10 min and centrifuged at 11000 rpm for 5 min. A 25 *μ*L aliquot of the homogenized samples was transferred into an Eppendorf tube and added with 20 *μ*L of internal standard (500 ng/mL voriconazole) followed by the addition of 100 *μ*L of methanol. After centrifugation at 11000 rpm for 5 min, a 5 *μ*L aliquot (plasma or brain) was injected in a LC/MS/MS system (Shimadzu LC-20A HPLC system; AnaShiseido, Tokyo, Japan) coupled with a TSQ Quantum Vantage triple quadrupole mass spectrometer equipped with a HESI source (ThermoFisher, San Jose, CA, USA). Chromatographic conditions were the following: guard column SecurityGuard C_18_ column (4 mm × 3.0 mm I.D., 5 μm, Phenomenex, Torrance, CA, USA), analytical column SB C_18_ (150 mm × 4.6 mm I.D., 5 μm, Agilent, USA), buffers 0.1% formic acid in 10 mM ammonium acetate/0.1% formic acid in methanol (10:90), flow rate 0.6 mL/min. Mass spectrometric conditions were the following: source HESI, scan mode SRM, polarity positive, vaporizer temperature 420 °C, ion sweep gas pressure 1 bar, auxiliary gas pressure 5 bar, capillary temperature 320 °C. Calibration curves were prepared in heparinized blank mice plasma (3–3000 ng/mL) or blank brain homogenate (1–10000 ng/g). Concentrations were calculated using a weighted least-squares linear regression (W = 1/x^2^). The linear regression equations of the calibration curves are presented in the Table 4.

The main pharmacokinetic parameters were calculated by non-compartmental analysis using Phoenix 1.3 (Pharsight USA).

AUC_0-t_: the area under the plasma/brain concentration versus time curve from time 0 to the last measurable concentration was calculated by using the linear trapezoidal rule.

C_max_: the maximum observed plasma concentration.

T_max_: the time corresponding to C_max_.

t_1/2_: the elimination half-life was calculated as 0.693/λ_z_. λ_z_ was obtained by log-linear regression using the terminal points of the plasma concentration-time curve.

### Behavioural analysis

#### Drug preparation for behavioural testing

For all behavioural analysis, GEBR-32a was dissolved in dimethylsulfoxide (DMSO) and stored at 4 °C, this stock solution was used for further dilutions in 0.5% methylcellulose. The vehicle condition received 0.5% methylcellulose with 0.005% DMSO, and all drug solutions had a fixed percentage of 0.5% methylcellulose and 0.005% DMSO.

#### Object Location Task (OLT)

The OLT has been derived from the Object Recognition Task (ORT)^54^. The apparatus consisted of a circular arena, 40 cm in diameter. The back half of the 40-cm high wall was made of grey polyvinyl chloride (PVC) and the front was made of transparent PVC. Fluorescent red tubes and a light bulb provided a constant illumination of about 20 lux on the floor of the apparatus. Two objects were placed in symmetrical positions at the mid-line between the black and transparent halves of the arena, about 5 cm away from wall. Four sets of 2 identical objects were used and these objects were presented to the animals in a balanced manner to avoid learning or place biases. The rodents were unable to displace the objects. Prior to the trials, mice were put in an empty cage for 4 minutes to increase arousal during testing. A test session comprised two 4-min trials. During the learning trial T1, the apparatus contained two identical objects placed on a horizontal line in the middle of the arena (object a1 and a2). The animals were always introduced into the apparatus with their nose towards the transparent wall segment (i.e. facing outwards to the front of the arena). Subsequently, the rodents were put back in their home cage for the inter-trial interval. After the retention interval, the animals were put back into the arena for the test trial T2, in which one object (a3) was in the same position as during T1 and the other (b) was moved to a different position to the front or back of the arena. The time spent exploring each object during T1 and T2 was recorded manually on a personal computer. Exploration was defined in the following manner: directing the nose to the object at a distance of no more than 2 cm and/or touching the object with the nose. Sitting on the object was not considered as exploratory behaviour. In order to avoid the presence of olfactory cues, the objects were thoroughly cleaned with a 70% ethanol solution before each trial. Prior to compound testing, animals were handled for 5 minutes on 3 consecutive days and allowed to explore the arena for 5 min. Subsequently, animals were accustomed to the complete OLT testing procedure including injections prior or after testing. In a first set of experiments, GEBR-32a (0.0003–0.01 mg/kg s.c.) was evaluated in adult WT mice with a 24 h inter-trial interval and was administered 3 h after the first trial (T1) of the OLT^40^,^55^. Afterwards, it was tested (0.001–0.3 mg/kg s.c.) in memory deficit models (aged WT and Tg2576 mice) with a 1 hour inter-trial interval and was administered 30 min before the first trial (T1) of the OLT. Of note, drug conditions were tested in semi-random order, so within one testing session, multiple treatment conditions were tested. The experimenter was blinded to the conditions that were being tested. Similar to the ORT, the readout parameters of the OLT are the time that rodents spent on exploring each object during T1 and T2. The exploration time (in seconds) of each object during T1 are presented as ‘a1’ and ‘a2’. The time spent in exploring the familiar and the moved objects in T2 are represented as ‘a3’ and ‘b’, respectively. Using these data, the following parameters were calculated: e1 = a1 + a2; e2 = a3 + b; d2 = (b–a3)/e2. The d2 index is a relative measure of discrimination that corrects for exploratory activity^56^. The d2 index can range from -1 to 1. A significant positive difference from zero indicates that the mice remembered the object locations from T1. Of note, mice require a minimum amount of exploration in order to show reliable memory performance^57^. Therefore, animals were removed from the analysis if they spent less than 9 s exploring the objects during T1 or T2. Normal distribution of data was evaluated by Kolmogorov–Smirnov and one-way ANOVA was performed to evaluate differences between the conditions. A one sample t-test was used to compare the d2 index of the conditions to zero (i.e. chance level).

#### Y-maze continuous alternation task

The apparatus was made of grey Plexiglas with three arms symmetrically placed together at a 120° angle. At the beginning of the trial, each mouse was placed in one of three arms (randomly divided and balanced) and was then allowed to freely explore the apparatus for 6 minutes. The number of entries into a different arm and the order was measured. An entry was only counted if all four paws of the animal were placed completely inside the arm. When a mouse visited all 3 arms consecutively, it made a triad. In between trials, the apparatus was cleaned with a 70% ethanol solution to avoid olfactory cues. In a first set of experiments, GEBR-32a (0.0003–0.3 mg/kg s.c.) was administered acutely to aged WT and Tg2576 mice 30 min before the trial. In a second set of experiments, GEBR-32a was administered chronically (23 days) at the dose of 0.003 mg/kg s.c. In order to reduce the number of animals necessary for experimentation and to increase statistical power, two testing sessions were performed (at day 22 and day 23) and combined. Of note, there was no statistical difference between testing sessions (day effect; data not shown) and, therefore, both test sessions were pooled. To measure spatial working memory, the percentage of alternations was calculated by taking the number of triads and dividing it through the maximum possible alternations (total entries minus 2) and multiply this by 100. A score of 50% alternations is considered chance level and, therefore, a significant difference from 50% is indicative of functional working memory. Normal distribution of data was evaluated by Kolmogorov–Smirnov and one-way ANOVA was performed to evaluate differences between the conditions. For each condition, one-sample t-test was used for comparison versus the score of 50%.

#### Ketamine/xylazine induced α_2_-adrenoceptor-mediated anaesthesia test

To investigate possible emetic-like effect of GEBR-32a, mice were anesthetized with of 60 mg/kg ketamine and 10 mg/kg xylazine (i.p. injection, 1 ml/kg). GEBR-32a (0.003–3 mg/kg) or vehicle were administered 15 minutes after the induction of anaesthesia and mice were put in dorsal position^26^. The outcome measurement was the total time the animals were anesthetized, measured from anaesthesia induction to the righting reflex (i.e. when mice were back on all four paws). In order to reduce the number of animals for experimentation and to get sufficient group sizes, the experiments were performed three times with 2 recovery days in between. Each animal did not receive the same condition more than once, i.e per test day the drug treatments were balanced over the animals so each condition was tested in every animal eventually. Normal distribution of data was evaluated by Kolmogorov–Smirnov and one-way ANOVA was used to compare all conditions.

### Electrophysiology recordings

Electrophysiological recordings have been carried out as previously described^48^. Briefly, hippocampal slices (400 μM thick) were obtained from aged WT and Tg2576 mice, chronically treated either with vehicle or with GEBR-32a (0.03 mg/kg s.c.), by means of a tissue chopper and subsequently were maintained under perfusion (1–3 ml/min) with saline solution (in mM: 124 NaCl, 4.4 KCl, 1.0 Na_2_HPO_4_, 25.0 NaHCO_3_, 2.0 CaCl_2_, 2.0 MgSO_4_,10 glucose; continuously aerated with 95% O2 and 5% CO2) in an interface chamber at 29 °C for 90 min prior to recording. A concentric bipolar platinum-iridium stimulation electrode and a low-resistance glass recording microelectrode filled with saline solution (5 mΩ resistance) were placed in CA1 stratum radiatum to record the extracellular field excitatory postsynaptic potential (fEPSP). Basal synaptic transmission was assayed by plotting the stimulus voltages against slopes of fEPSP. For LTP experiments, a 15-min baseline was recorded every min at an intensity that evoked a response ~35% of the maximum response. LTP was induced using a θ-burst stimulation, consisting of 4 pulses at 100 Hz, with the bursts repeated at 5 Hz and each tetanus including three 10-burst trains separated by 15 s [33].

Normal distribution of data was evaluated by Kolmogorov–Smirnov test and two-way ANOVA was used to determine the rescuing effect of GEBR-32a on synaptic dysfunction.

## Additional Information

**How to cite this article**: Ricciarelli, R. *et al*. Memory-enhancing effects of GEBR-32a, a new PDE4D inhibitor holding promise for the treatment of Alzheimer’s disease. *Sci. Rep.* **7**, 46320; doi: 10.1038/srep46320 (2017).

**Publisher's note:** Springer Nature remains neutral with regard to jurisdictional claims in published maps and institutional affiliations.

## Figures and Tables

**Table 1:** Enzymatic profile of GEBR-32a.

PDE	GEBR-32a % inhibition	Comparator % inhibition
PDE1B	10.7	74.4 (Sildenafil 10 μM)
PDE2A3	NI	89.6 (BAY 60-7550 1 nM)
PDE4A1	41.0	66.5 (Rolipram 10 μM)
PDE4A4	11.0	60.1 (Rolipram 10 μM)
PDE4B1	41.6	78.6 (Rolipram 10 μM)
PDE4B2	26.3	69.6 (Rolipram 10 μM)
PDE4B3	39.4	68.6 (Rolipram 10 μM)
**PDE4D1**	**82**.**2**	83.1 (Rolipram 1 μM)
**PDE4D2**	**87**.**8**	70.8 (Rolipram 1 μM)
**PDE4D3**	**63**.**6**	52.6 (Rolipram 1 μM)
**PDE4D5**	**67**.**3**	58.0 (Rolipram 1 μM)
**PDE4D7**	**90**.**7**	86.7 (Rolipram 1 μM)
PDE5A1	4.97	74.9 (Sildenafil 100 nM)
PDE7A	NI	78.6 (BRL-50481 10 μM)
PDE7B	8.61	47.3 (Dipyridamole 50 μM)
PDE8A1	NI	64.4 (Dipyridamole 50 μM)
PDE8B1	23.5	57.4 (Dipyridamole 50 μM)
PDE9A1	NI	92.7 (SB 36216 1 μM)
PDE10A1	NI	90.7 (Papaverine 1 μM)
PDE11A1	NI	91.0 (Dipyridamole 10 μM)

Inhibitory activity of GEBR-32a has been evaluated at the concentration of 10 μM on 20 different recombinant human PDEs expressed in baculovirus. Percent inhibition of comparators (at appropriate concentrations) is also reported. NI = no inhibition. In bold, PDEs whose activity was inhibited more than 50% by GEBR-32a.

**Table 2:** GEBR-32a potency towards PDE4D isoforms.

PDE	GEBR-32a IC_50_ (μM)
PDE4D1	4.97
PDE4D2	2.89
PDE4D3	2.43
PDE4D5	3.18
PDE4D7	1.14

GEBR-32a has been tested at five different concentrations (1nM-100 μM) on human recombinant PDE4D isoforms expressed in baculovirus. IC_50s_ have been obtained by non-linear regression analysis of the concentration-inhibition curves.

**Table 3:** Main pharmacokinetic parameters of GEBR-32a.

Parameters	Plasma	Brain
C_max_ (ng/ml)	993	2608
T_max_ (h)	0.33	0.33
AUC_0-t_ (ng·h/ml)	861	2330
t_1/2_ (h)	0.95	0.95
AUC_0-t_ Ratio (Brain/Plasma)	2.71	

GEBR-32a was administered subcutaneously to mice at the dose of 10 mg/kg. Blood samples and brain tissues were collected at 7 different time points and analysed for GEBR-32a content. The main pharmacokinetic parameters were calculated by non-compartmental analysis. C_max_: the maximum observed plasma/brain concentration; T_max_: the time corresponding to C_max_; AUC_0-t_: the area under the plasma/brain concentration versus time curve from time 0 to the last time point; t_1/2_: elimination half-life.

**Table 4:**
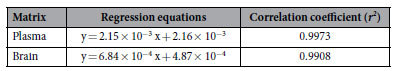
Calibration curves for GEBR-32a HPLC analysis.

## References

[b1] Kandel E. R. The molecular biology of memory: cAMP, PKA, CRE, CREB-1, CREB-2, and CPEB. Mol. Brain. 5, 14 (2012). 22583753 10.1186/1756-6606-5-14 PMC3514210

[b2] Bliss T. V. & Lømo T. Long-lasting potentiation of synaptic transmission in the dentate area of the anaesthetized rabbit following stimulation of the perforant pathway. J. Physiol. 232, 331–356 (1973). 4727084 10.1113/jphysiol.1973.sp010273 PMC1350458

[b3] Wong S. T. . Calcium-stimulated adenylyl cyclase activity is critical for hippocampus dependent long-term memory and late phase LTP. Neuron. 23, 787–798 (1999). 10482244 10.1016/s0896-6273(01)80036-2

[b4] Wang H., Ferguson G. D., Pineda V. V., Cundiff P. E. & Storm D. R. Overexpression of type-1 adenylyl cyclase in mouse forebrain enhances recognition memory and LTP. Nat. Neurosci. 7, 635–642 (2004). 15133516 10.1038/nn1248

[b5] Zhang M. & Wang H. Mice overexpressing type 1 adenylyl cyclase show enhanced spatial memory flexibility in the absence of intact synaptic long-term depression. Learn. Mem. 20, 352–367 (2013). 23772089 10.1101/lm.030114.112 PMC3687257

[b6] Frey U., Huang Y. Y. & Kandel E. R. Effects of cAMP simulate a latestage of LTP in hippocampal neurons. Science. 260, 1661–1664 (1993). 8389057 10.1126/science.8389057

[b7] Huang Y. Y. & Kandel E. R. Recruitment of long-lasting and protein kinase A-dependent long-term potentiation in the CA1 region of hippocampus requires repeated tetanization. Learn Mem. 1, 74–82 (1994). 10467587

[b8] Abel T. . Genetic demonstration of a role for PKA in the late phase of LTP and in hippocampus-based long-term memory. Cell. 88, 615–626 (1997). 9054501 10.1016/s0092-8674(00)81904-2

[b9] Barco A., Alarcon J. M. & Kandel E. R. Expression of constitutively active CREB protein facilitates the late phase of long-term potentiation by enhancing synaptic capture. Cell. 108, 689–703 (2002). 11893339 10.1016/s0092-8674(02)00657-8

[b10] Koh M. T., Thiele T. E. & Bernstein I. L. Inhibition of protein kinase A activity interferes with lon-term, but not short-term, memory of conditioned taste aversions. Behav. Neurosci. 116, 1070–1074 (2002). 12492305

[b11] Pittenger C. . Reversible inhibition of CREB/ATF transcription factors in region CA1 of the dorsal hippocampus disrupts hippocampus-dependent spatial memory. Neuron. 34, 447–462 (2002). 11988175 10.1016/s0896-6273(02)00684-0

[b12] Young J. Z., Isiegas C., Abel T. & Nguyen P. V. Metaplasticity of the late phase of long-term potentiation: a critical role for protein kinase A in synaptic tagging. Eur. J. Neurosci. 23, 1784–1794 (2006). 16623835 10.1111/j.1460-9568.2006.04707.x PMC2921966

[b13] Gelinas J. N. . Activation of exchange protein activated by cyclic-AMP enhances long-lasting synaptic potentiation in the hippocampus. Learn. Mem. 15, 403–411 (2008). 18509114 10.1101/lm.830008 PMC2414251

[b14] Ma N., Abel T. & Hernadez P. J. Exchange protein activated by cAMP enhances long-term memory formation independent of protein kinase A. Learn. Mem. 16, 367–370 (2009). 19470652 10.1101/lm.1231009 PMC2704102

[b15] Suzuki A. . Upregulation of CREB-mediated transcription enhances both short- and long-term memory. J. Neurosci. 31, 8786–8802 (2011). 21677163 10.1523/JNEUROSCI.3257-10.2011 PMC6622960

[b16] Kida S. A functional role for CREB as a positive regulator of memory formation and LTP. Exp. Neurobiol. 21, 136–140 (2012). 23319873 10.5607/en.2012.21.4.136 PMC3538177

[b17] Bollen E. . Improved long-term memory via enhancing cGMP-PKG signaling requires cAMP-PKA signaling. Neuropsychopharmacology. 39, 2497–2505 (2014). 24813825 10.1038/npp.2014.106 PMC4207334

[b18] Richter W., Menniti F. S., Zhang H. T. & Conti M. PDE4 as target for cognition enhancement. Expert Opin. Ther. Targets. 17, 1011–1027 (2013). 23883342 10.1517/14728222.2013.818656 PMC4066988

[b19] Hansen R. T. III & Zhang H. T. Phosphodiesterase-4 modulation as potential therapeutic for cognitive loss in pathological and nonpathological aging: possibilities and pitfalls. Curr. Pharm. Des. 21, 291–302 (2015). 25159075 10.2174/1381612820666140826114208

[b20] Heckman P. R., Blokland A., Ramaekers J. & Prickaerts J. Phosphodiesterase inhibitors as a target for cognition enhancement in aging and Alzheimer’s disease: a translational overview. Curr. Pharm. Des. 21, 317–331 (2015). 25159073 10.2174/1381612820666140826114601

[b21] Gurney M. E., D’Amato E. C. & Burgin A. B. Phosphodiesterase-4 (PDE4) molecular pharmacology and Alzheimer’s disease. Neurotherapeutics. 12, 49–56 (2015). 25371167 10.1007/s13311-014-0309-7 PMC4322084

[b22] Heckman P. R., Blokland A., Ramaekers J. & Prickaerts J. PDE and cognitive processing: beyond the memory domain. Neurobiol. Learn. Mem. 119, 108–122 (2015). 25464010 10.1016/j.nlm.2014.10.011

[b23] Hebenstreit G. F. . Rolipram in major depressive disorder: results of a double-blind comparative study with imipramine. Pharmacopsychiatry. 22, 156–160 (1989). 2668980 10.1055/s-2007-1014599

[b24] Ricciarelli R. & Fedele E. Phosphodiesterase 4D: an enzyme to remember. Br. J. Pharmacol. 172, 4785–4789 (2015). 26211680 10.1111/bph.13257 PMC4621991

[b25] Bruno O. . New Selective phosphodiesterase 4D inhibitors differently acting on long, short, and supershort isoforms. J. Med. Chem. 52, 6546–6557 (2009). 19827751 10.1021/jm900977c

[b26] Bruno O. . GEBR-7b, a novel PDE4D selective inhibitor that improves memory in rodents at non-emetic doses. Br. J. Pharmacol. 164, 2054–2063 (2011). 21649644 10.1111/j.1476-5381.2011.01524.x PMC3246667

[b27] Brullo C. . Synthesis, biological evaluation, and molecular modeling of new 3-(cyclopentyloxy)-4-methoxybenzaldehyde O-(2-(2,6-dimethylmorpholino)-2-oxoethyl) oxime (GEBR-7b) related phosphodiesterase 4D (PDE4D) inhibitors. J. Med. Chem. 57, 7061–7072 (2014). 25126889 10.1021/jm500855w

[b28] Sierksma A. S. . Improvement of spatial memory function in APPswe/PS1dE9 mice after chronic inhibition of phosphodiesterase type 4D. Neuropharmacology. 77, 120–130 (2014). 24067928 10.1016/j.neuropharm.2013.09.015

[b29] Brullo C. . Synthesis, biological activities and pharmacokinetic properties of new fluorinated derivatives of selective PDE4D inhibitors. Bioorg. Med. Chem. 23, 3426–3435 (2015). 25936260 10.1016/j.bmc.2015.04.027

[b30] Brullo C. . New insights into selective PDE4D inhibitors: 3-(Cyclopentyloxy)-4-methoxybenzaldehyde O-(2-(2,6-dimethylmorpholino)-2-oxoethyl) oxime (GEBR-7b) structural development and promising activities to restore memory impairment. Eur. J. Med. Chem. 124, 82–102 (2016). 27560284 10.1016/j.ejmech.2016.08.018

[b31] Müller K., Faeh C. & Diederich F. Fluorine in pharmaceuticals: looking beyond intuition. Science. 317, 1881–1886 (2007). 17901324 10.1126/science.1131943

[b32] Hagmann W. K. The many roles for fluorine in medicinal chemistry. J. Med. Chem. 51, 4359−4369 (2008). 18570365 10.1021/jm800219f

[b33] Park B. K., Kitteringham N. R. & O’Neill P. M. Metabolism of fluorine-containing drugs. Ann. Rev. Pharmacol. Toxicol. 41, 443−470 (2001). 11264465 10.1146/annurev.pharmtox.41.1.443

[b34] Eastman K. J., Gillis E. P. & Meanwell N. A. Tactical applications of fluorine in drug design and development. In Fluorine in Heterocyclic Chemistry(ed. Nenajdenko V.) Volume 1 1–54 (Springer International 2014).

[b35] Wang J. . Fluorine in pharmaceutical industry: fluorine-containing drugs introduced to the market in the last decade (2001−2011). Chem Rev. 114, 2432−2506 (2014). 24299176 10.1021/cr4002879

[b36] Krause W. & Kühne G. Pharmacokinetics of rolipram in the rhesus and cynomolougs monkey, the rat and the rabbit. Studies on species differences. Xenobiotica. 18, 561–571 (1988). 3400274 10.3109/00498258809041693

[b37] Vanmierlo T. . The PDE4 inhibitor roflumilast improves memory in rodents at non-emetic doses. Behav. Brain Res. 303, 26–33 (2016). 26794595 10.1016/j.bbr.2016.01.031

[b38] Burgin A. N. . Design of phosphodiesterase 4D (PDE4D) allosteric modulators for enhancing cognition with improved safety. Nat. Biotechnol. 28, 63–70 (2010). 20037581 10.1038/nbt.1598

[b39] Sutcliffe J. S. . Efficacy of selective PDE4D negative allosteric modulators in the object retrieval task in female cynomolgus monkeys (Macaca fascicularis). PLoS One. 9, e102449 (2014). 25050979 10.1371/journal.pone.0102449 PMC4106781

[b40] Rutten K. . Time-dependent involvement of cAMP and cGMP in consolidation of object memory: studies using selective phosphodiesterase type 2, 4 and 5 inhibitors. Eur. J. Pharmacol. 558, 107–112 (2007). 17207788 10.1016/j.ejphar.2006.11.041

[b41] Hsiao K. . Correlative memory deficits, Abeta elevation, and amyloid plaques in transgenic mice. Science 274, 99–102 (1996). 8810256 10.1126/science.274.5284.99

[b42] Vitolo O. V. . Amyloid beta-peptide inhibition of the PKA/CREB pathway and long-term potentiation: reversibility by drugs that enhance cAMP signaling. Proc. Natl. Acad. Sci. USA 99, 13217–13221 (2002). 12244210 10.1073/pnas.172504199 PMC130613

[b43] Wiescholleck V. & Manahan-Vaughan D. PDE4 inhibition enhances hippocampal synaptic plasticity *in vivo* and rescues MK801-induced impairment of long-term potentiation and object recognition memory in an animal model of psychosis. Transl. Psychiatry. 2, e89 (2012). 22832854 10.1038/tp.2012.17 PMC3309535

[b44] Sanderson T. M. & Sher E. The role of phosphodiesterases in hippocampal synaptic plasticity. Neuropharmacology. 74, 86–95 (2013). 23357335 10.1016/j.neuropharm.2013.01.011

[b45] Robichaud A. . Assessing the emetic potential of PDE4 inhibitors in rats. Br. J. Pharmacol. 135, 113–118 (2002). 11786486 10.1038/sj.bjp.0704457 PMC1573119

[b46] Robichaud A., Savoie C., Stamatiou P. B., Tattersall F. D. & Chan C. C. PDE4 inhibitors induce emesis in ferrets via a noradrenergic pathway. Neuropharmacology. 40, 262–269 (2001). 11114405 10.1016/s0028-3908(00)00142-8

[b47] McLachlan C. S. . Changes in PDE4D isoforms in the hippocampus of a patient with advanced Alzheimer disease. Arch. Neurol. 64, 456–457 (2007). 17353396 10.1001/archneur.64.3.456

[b48] Gong B. . Persistent improvement in synaptic and cognitive functions in an Alzheimer mouse model after rolipram treatment. J. Clin. Invest. 114, 1624–1634 (2004). 15578094 10.1172/JCI22831 PMC529285

[b49] Kraft P. . The phosphodiesterase-4 inhibitor rolipram protects from ischemic stroke in mice bt reducing bllod-brain-barrier damage, inflammation and thrombosis. Exp. Neurol. 247, 80–90 (2013). 23570902 10.1016/j.expneurol.2013.03.026

[b50] Caraci F. . A key role for TGF-b1 in hippocampal synaptic plasticity and memory. Sci. Rep. 5, 11252 (2016). 10.1038/srep11252 PMC4462026 26059637

[b51] Guo H. . FFPM, a PDE4 inhibitor, reverses learning and memory deficits in APP/PS1 transgenic mice via cAMP/PKA/CREB signalling and anti-inflammatory effects. Neuropharmacology 116, 260–269 (2017). 28065587 10.1016/j.neuropharm.2017.01.004

[b52] Marte A. . Alterations of glutamate release in the spinal cord of mice with experimental autoimmune encephalomyelitis. J. Neurochem. 115, 343–352 (2010). 20649849 10.1111/j.1471-4159.2010.06923.x

[b53] d’Abramo C., Ricciarelli R., Pronzato M. A. & Davis P. Troglitazone, a perixosome proliferator-activated receptor-gamma agonist, decreases tau phosphorylation in CHOtau4R cells. J. Neurochem. 98, 1068–1077 (2006). 16787414 10.1111/j.1471-4159.2006.03931.x

[b54] Ennaceur A. & Delacour J. A new one-trial test for neurobiological studies of memory in rat. 1:Behavioral data. Behav. Brain Res. 31:47–59 (1988). 3228475 10.1016/0166-4328(88)90157-x

[b55] Rutten K. . Phosphodiesterase inhibitors enhance object memory independent of cerebral blood flow and glucose utilization in rats. Neuropsychopharmacology. 34, 1914–1925 (2009). 19262466 10.1038/npp.2009.24

[b56] Akkerman S. . Object recognition testing: Methodological considerations on exploration and discrimination measures. Behav. Brain Res. 232, 335–347 (2012). 22490364 10.1016/j.bbr.2012.03.022

[b57] Akkerman S., Prickaerts J., Steinbusch H. W. M. & Blokland A. Object recognition testing: Statistical considerations. Behav. Brain Res. 232, 317–322 (2012). 22481080 10.1016/j.bbr.2012.03.024

